# Complete Genome Assembly and Annotation of Escherichia coli Bacteriophage 107

**DOI:** 10.1128/mra.00106-23

**Published:** 2023-05-16

**Authors:** Joshua C. Schwarz, Benjamin K. Chan, Paul E. Turner, Alita R. Burmeister

**Affiliations:** a Department of Ecology and Evolutionary Biology, Yale University, New Haven, Connecticut, USA; b BEACON Center for the Study of Evolution in Action, East Lansing, Michigan, USA; c Program in Microbiology, Yale School of Medicine, New Haven, Connecticut, USA; d Center for Phage Biology and Therapy, Yale University, New Haven, Connecticut, USA; Portland State University

## Abstract

We present the annotated genome sequence of Escherichia coli bacteriophage 107, a T4-like bacteriophage. Phage 107 has a genome length of 167,509 bp and 287 predicted genes.

## ANNOUNCEMENT

Wastewater environments are sources of potentially therapeutic phages (1). Bacteriophage 107 was isolated from influent wastewater at the East Shore Water Pollution Abatement Facility (New Haven, CT) after aerobic enrichment at 37°C on Escherichia coli BW25113 for 24 h in tryptic soy broth. The phage was isolated by plaque assay and then plaque purified three times prior to amplification.

DNA was isolated from the phage lysate using a Norgen phage DNA isolation kit (46850) and sequenced at MiGS (Pittsburgh, PA). Libraries were prepared using an Illumina DNA prep kit and IDT for Illumina 10-bp indices and sequenced on an Illumina NextSeq 2000 instrument, producing 2 × 151-bp reads. The raw data were demultiplexed and adapters removed using bcl2fastq v.2.20.0.422 (2), resulting in 1,459,698 reads.

We assembled and annotated the genomic data using Galaxy (3) and Apollo (4), hosted at the Center for Phage Technology (Texas A&M University). The reads were subsetted using the FASTA subset tool to a target coverage of 200×. FastQC v.0.72+galaxy1 (5) was run with default parameters to assess the sequence quality. Trim sequences v.1.0.2+galaxy0 (6) was used to set a quality threshold of 30, and FastQC v.0.72+galaxy1 was run on the trimmed reads to confirm trimming.

We then used SPAdes v.3.12.0+galaxy1 to assemble the reads, producing 482 contigs. Of these, 481 contigs had less than 5% genome coverage or were single polynucleotide repeats and were omitted from further analysis. The one remaining contig of 167,509 bp had a coverage of 149× and included a 55-bp terminal overlap, indicating a circular genome. These 55 bp were manually removed.

Galaxy’s Structural Annotation PAP Workflow v2021.02 was used to predict potential genes using GLIMMER3 v.0.2 (7), MetaGeneAnnotator v.1.0.0 (8), and sixpack v.5.0.0+galaxy2 (9). Using NCBI’s BLASTn (10), it was determined that phage 107 is related to phage T4 (GenBank accession number MT984581.1), with 94.7% identity and 88% query coverage. Reopen FASTA Sequences (Galaxy v.2.0) (11) was used to reopen the genome between predicted open reading frames (ORFs) and to provide synteny with T4. We then reran the PAP workflow on the reopened genome. The predicted genes were manually confirmed based on Shine-Dalgarno sequences, start and stop codons, and the overlaps or distances between the predicted genes.

Galaxy’s Functional Annotation PAP Workflow v.2022.01 (11) was used to generate comparative evidence from three databases (Canonical Phages, Swiss-Prot, and Nonredundant Phages) to predict gene functions. Predicted gene calls were individually analyzed and manually annotated. InterProScan v.5.52-86.0 (12) and TMHMM v.1.0.2 (13) were used to resolve conflicting evidence. EDGE bioinformatics (14) was used to determine that phage 107 has a GC content of 35.36% and contains no known virulence factors or toxins. The final annotation contained 287 annotated genes, including 125 ORFs with putative functions and 11 putative tRNA genes.

### Data availability.

The genome is available at NCBI GenBank under accession number OQ599309.1. The sequences are available in the Sequence Read Archive (SRA) under accession number SRX19352635 and BioProject accession number PRJNA934296. For more robust identification, we also used “RGB-005” as a unique internal identifier for the bacteriophage 107 isolate.
